# Host–Microbe Mechanisms of *Fusobacterium nucleatum* in Colorectal Cancer

**DOI:** 10.1111/omi.70038

**Published:** 2026-08-19

**Authors:** Hadda Lyzandra Austríaco Leite, Fernanda Ferreira Lopes

**Affiliations:** ^1^ Postgraduate Program in Dentistry Federal University of Maranhão São Luís Brazil

**Keywords:** colorectal cancer, *fusobacterium nucleatum*, host–microbe interactions, metastasis, molecular mechanisms, tumor microenvironment

## Abstract

The oral‐derived pathobiont *Fusobacterium nucleatum* has increasingly been implicated in colorectal cancer (CRC) progression through its ability to modulate inflammatory, immunological, and molecular pathways. *F. nucleatum* has emerged as a key oral‐derived pathobiont frequently associated with tumor aggressiveness and alterations in the colorectal tumor microenvironment. This review critically examines recent mechanistic evidence from in vitro, in vivo, and advanced experimental models to elucidate how *F. nucleatum* interacts with host pathways involved in colorectal carcinogenesis. Available evidence indicates that this bacterium actively reshapes the tumor microenvironment through coordinated effects on immune signaling, epigenetic regulation, metabolic adaptation, and metastatic competence. Mechanistically, *F. nucleatum* promotes sustained inflammatory activation, macrophage polarization, epithelial–mesenchymal transition, endothelial remodeling, and resistance to regulated cell death pathways, while also influencing transcriptional and oncogenic programs associated with tumor progression. Emerging evidence further suggests that microbial effects may be modulated by tumor heterogeneity and long‐term environmental exposures, reinforcing the complexity of host–microbiome interactions in CRC. Collectively, the findings support the concept that *F. nucleatum* functions as an active biological driver rather than a passive microbial bystander in CRC progression. These insights highlight the translational relevance of tumor‐associated microbiota and support the need for approaches combining mechanistic, microbial, and clinical data to refine precision strategies at the tumor–microbiome interface.

## Graphical abstract

*Fusobacterium nucleatum* and colorectal cancer.

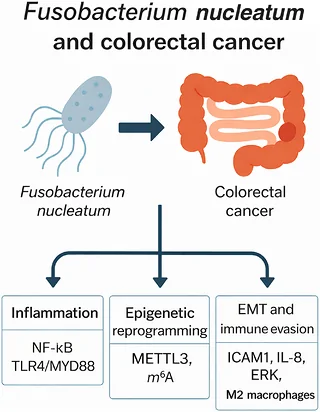

## 1 Introduction

The human oral cavity harbors a highly diverse and spatially organized microbiota composed of bacteria, fungi, archaea, protozoa, and viruses. These microorganisms inhabit distinct oral niches and form complex communities that interact continuously with host epithelial and immune systems. Under homeostatic conditions, these interactions contribute to colonization resistance and oral health; however, ecological disruption may promote dysbiosis, persistent inflammation, and the development of oral and systemic diseases (Baker et al. 2024).

Within this microbial ecosystem, the genus *Fusobacterium* comprises Gram‐negative, obligately anaerobic bacteria that colonize human mucosal surfaces, with the oral cavity representing their principal ecological reservoir. The genus includes several species recovered from the oral cavity in addition to *Fusobacterium nucleatum*, such as *Fusobacterium periodonticum* and *F. necrophorum*, which differ in their ecological niches and disease associations. *F. periodonticum* is phylogenetically closely related to *F. nucleatum* and is similarly implicated in periodontal biofilm formation and periodontitis, whereas *F. necrophorum* is associated with more invasive and destructive forms of periodontal and pharyngeal disease, particularly in immunocompromised individuals, and can give rise to severe extraoral infections such as Lemierre syndrome (Brennan and Garrett 2019; Riordan 2007). Among oral fusobacteria, *F. nucleatum* is one of the most frequently detected species in dental plaque and is found at both healthy and diseased sites. Its biological behavior is therefore context‐dependent: it may participate in polymicrobial homeostasis at low abundance but can act as an opportunistic pathobiont when ecological conditions favor dysbiosis and inflammation (Brennan and Garrett 2019; Krieger et al. 2024).

In oral biofilms, *F. nucleatum* occupies a central ecological position because of its broad capacity for interspecies coaggregation. It acts as a bridging organism between early colonizers, particularly oral streptococci, and later, predominantly anaerobic colonizers such as *Porphyromonas gingivalis* and *Aggregatibacter actinomycetemcomitans*. This bridging function contributes to microbial succession, biofilm maturation, and the structural organization of dental plaque. These interactions are mediated by several surface‐associated proteins and adhesins, including RadD, FadA, and Fap2, which participate in bacterial coaggregation, epithelial adhesion, host‐cell invasion, and immune modulation (Brennan and Garrett 2019; Groeger et al. 2022; Chen et al. 2022).

The biological relevance of *F. nucleatum* extends beyond biofilm architecture. The bacterium is enriched in gingivitis and periodontitis and can adhere to and invade gingival epithelial cells, interact with host pattern‐recognition receptors, and stimulate inflammatory mediators and antimicrobial responses. Infection of oral epithelial cells has been associated with activation of intracellular signaling pathways, including NF‐κB, and with increased production of cytokines and chemokines such as IL‐6, IL‐8, IL‐1β, and TNF‐α. Through these effects, *F. nucleatum* may promote epithelial barrier alteration, inflammatory cell recruitment, immune‐response modulation, and persistence of the dysbiotic periodontal microenvironment (Groeger et al. 2022; Chen et al. 2022).

These ecological and pathogenic properties are not necessarily restricted to the oral cavity. The oral and intestinal microbiotas are connected through continuous microbial dispersal, particularly by swallowing and, under inflammatory conditions, possibly through hematogenous dissemination. Although the healthy intestinal microbiota generally restricts persistent colonization by oral microorganisms, disturbances in microbial or epithelial homeostasis may facilitate their ectopic establishment. Oral pathobionts may consequently contribute to extraoral disease by promoting inflammation, modifying immune responses, interacting with resident microbial communities, and disrupting epithelial barrier function (Kunath et al. 2024).

Colorectal cancer (CRC) provides a particularly relevant model through which to examine this oral–gut connection. *F. nucleatum*, although common in the oral cavity and uncommon in the lower gastrointestinal tract of healthy individuals, is consistently enriched in a subset of colorectal tumors. Importantly, several properties implicated in its oral pathogenicity are recapitulated within the colorectal tumor microenvironment, including epithelial adhesion and invasion, biofilm‐associated persistence, inflammatory signaling, immune evasion, epithelial barrier disruption, and modulation of host‐cell survival pathways. These mechanistic parallels provide a strong conceptual basis for examining CRC‐associated *F. nucleatum* through an oral microbiology perspective rather than regarding it solely as a component of the intestinal microbiome (Brennan and Garrett 2019; Kunath et al. 2024; Zepeda‐Rivera et al. 2024).

The species should also not be regarded as a biologically homogeneous entity. *F. nucleatum* has traditionally been divided into the subspecies *nucleatum*, *animalis*, *vincentii*, and *polymorphum*, which differ in genomic composition, ecological distribution, host associations, and potentially pathogenic behavior (Krieger et al. 2024). Recent phylogenomic evidence has further demonstrated that colorectal tumor‐associated strains are predominantly affiliated with *F. nucleatum* subsp. *animalis*. Within this subspecies, the Fna C2 clade is specifically enriched in CRC tissue and carries genetic traits associated with enhanced gastrointestinal colonization, metabolic adaptability, and tumor‐promoting potential (Zepeda‐Rivera et al. 2024). These findings indicate that cancer‐associated properties cannot necessarily be generalized to all *F. nucleatum* strains and highlight the importance of subspecies‐ and clade‐level identification when interpreting experimental evidence.

Against this background, a comprehensive understanding of the host–microbe mechanisms through which *F. nucleatum* influences CRC biology is particularly relevant to an oral microbiology audience. Accordingly, this review critically synthesizes mechanistic evidence derived from CRC cell lines, immune‐cell cultures, human tissue analyses, murine xenograft and metastasis models, and other experimental platforms. Particular emphasis is placed on how biological functions established in the oral niche are reproduced or adapted within the colorectal tumor microenvironment to influence inflammatory signaling, immune regulation, epigenetic and transcriptional control, metabolic adaptation, invasion, metastatic dissemination, and therapeutic resistance.

## 2 Literature Search and Evidence Integration

This review was developed through a critical evaluation of recent experimental literature investigating the molecular and cellular mechanisms through which *F. nucleatum* modulates CRC biology. Particular emphasis was placed on mechanistic evidence derived from in vitro systems, in vivo models, tumor organoids, and co‐culture platforms, with the aim of identifying convergent host–microbe signaling pathways and biological processes relevant to colorectal tumor progression.

The literature considered in this review included experimental studies examining the effects of *F. nucleatum* or its virulence‐associated components on key mechanisms implicated in colorectal carcinogenesis. These mechanisms encompassed inflammatory and oncogenic signaling pathways (including NF‐κB, Wnt/β‐catenin, and Toll‐like receptor–associated signaling), immune modulation, epigenetic and transcriptional regulation, metabolic reprogramming, regulated cell death pathways, and cellular behaviors related to proliferation, invasion, metastasis, and therapeutic resistance. Purely descriptive microbiome studies, as well as clinical or epidemiological investigations lacking mechanistic experimental evaluation, were not considered, given the primary focus on molecular and cellular host–microbe interactions.

Rather than providing an exhaustive catalog of individual experiments, the evidence was synthesized thematically, prioritizing biological plausibility, mechanistic consistency across CRC cell lines, murine models, tumor organoids, and co‐culture platforms, and the integration of findings into broader conceptual frameworks relevant to CRC progression and tumor–microbiome interactions.

## 3 Mechanistic Interactions of *F. nucleatum* in Colorectal Carcinogenesis

### 3.1 Strain Adaptation, Virulence, and Colorectal Niche Colonization

An important dimension for contextualizing the mechanistic evidence discussed in this review lies in recent advances in the taxonomy and genomics of *F. nucleatum*. Earlier interpretations frequently attributed virulence to the species as a whole; however, emerging genomic analyses indicate that CRC‐associated pathogenicity is largely driven by specific, evolutionarily adapted lineages. In particular, Zepeda‐Rivera et al. (2024) identified a genetically distinct clade, designated Fna C2, which is markedly enriched within the CRC niche and harbors an accessory genome that confers adaptive advantages for survival and persistence in the lower gastrointestinal tract.

The adaptive features of Fna C2 include enrichment of genes involved in 1,2‐propanediol (*pdu*) and ethanolamine (*eut*) metabolism, as well as the presence of a glutamate‐dependent acid resistance system (*gdar*), which collectively enhance bacterial fitness under the nutrient‐competitive and acidic conditions characteristic of the tumor microenvironment (Zepeda‐Rivera et al. 2024). These observations support the notion that CRC‐associated virulence is not a universal property of *F. nucleatum*, but rather a strain‐specific trait shaped by ecological selection, as also emphasized by Alon‐Maimon et al. (2022).

This phylogenetic stratification is critical for interpreting the pro‐tumorigenic mechanisms highlighted in this review, including NF‐κB activation, induction of epithelial–mesenchymal transition (EMT), and suppression of ferroptosis. Taken together, these findings reinforce the concept that strain‐level adaptation underlies the pathogenic potential of *F. nucleatum* in CRC, with direct implications for mechanistic interpretation, biomarker development, and the design of targeted microbiome‐based interventions.

Evidence also suggests a role for *F. nucleatum* in the early stages of colorectal carcinogenesis. Based on analyses of human adenoma and CRC biopsy specimens, Rezasoltani et al. (2025) found that increased expression of the FadA adhesin was associated with dysregulation of a competing endogenous RNA (ceRNA) network involving ANXA2, LINC00460, and microRNA let‐7a‐2. Although direct experimental manipulation was not performed in this study, these findings suggest that bacterial presence may influence transcriptional reprogramming during the adenoma–carcinoma transition.

Annexin (A2ANXA2) is a calcium‐dependent phospholipid‐binding protein involved in membrane organization, cytoskeletal remodeling, cell adhesion, and extracellular matrix interactions. Increased ANXA2 expression has been associated with enhanced tumor proliferation, invasion, angiogenesis, and metastatic dissemination in CRC (Huang et al. 2025). LINC00460 is a long non‐coding RNA (lncRNA) involved in the regulation of gene expression through ceRNA networks and has been implicated in CRC progression by promoting cell proliferation, migration, invasion, and EMT. In contrast, the let‐7 family of microRNAs generally acts as a tumor suppressor by negatively regulating multiple oncogenic pathways, and its downregulation has been associated with increased tumor aggressiveness. Alterations involving these regulatory molecules therefore suggest that *F. nucleatum* may contribute to colorectal carcinogenesis not only through inflammatory signaling but also through modulation of host transcriptional regulatory networks (Su et al. 2022; Rezasoltani et al. 2025).

Beyond intracellular signaling, the spatial organization of *F. nucleatum* into invasive biofilms within colorectal adenomas and carcinomas provides an additional layer of complexity (Queen et al. 2025). These structured microbial communities may contribute to early tissue invasion by promoting persistent bacterial adhesion to the epithelial surface, increasing local bacterial density, and facilitating prolonged interactions between bacterial adhesins and host cells. Biofilm formation may also impair epithelial barrier integrity, sustain chronic inflammatory signaling, and protect bacteria from host immune clearance, thereby creating a microenvironment conducive to persistent colonization and tumor progression (Brennan and Garrett 2019). Although the precise mechanisms remain incompletely understood, the presence of invasive biofilms in colorectal adenomas and carcinomas supports the hypothesis that microbial spatial organization, in addition to molecular signaling, contributes to early colorectal carcinogenesis (Queen et al. 2025).

### 3.2 Immune Modulation and Inflammatory Signaling

*F. nucleatum* actively reshapes the colorectal tumor immune microenvironment through coordinated interactions with host immune receptors and intracellular inflammatory signaling pathways. A recurring mechanistic theme across experimental studies is sustained activation of the NF‐κB pathway, a central regulator linking inflammation, immune modulation, and tumor cell survival. This activation appears to occur through both direct host–microbe interactions and bacterial metabolite–mediated signaling cascades.

Structural components of *F. nucleatum*, particularly lipopolysaccharide (LPS) and O‐antigen from the *animalis* subspecies, have been shown to directly interact with the Siglec‐7 receptor expressed on dendritic cells and macrophages, altering cytokine production and promoting phenotypes consistent with tumor‐associated macrophages (Lamprinaki et al. 2021). Using RAW264.7 macrophages cultured in vitro, Zheng et al. (2025) demonstrated that infection with *F. nucleatum* induces polarization toward an M2‐like phenotype through activation of NF‐κB and cytokine–receptor signaling pathways, reinforcing the establishment of an immunosuppressive and tumor‐permissive microenvironment (Zheng et al. 2025).

Using CRC cell lines exposed to bacterial supernatants and purified ADP‐heptose, Martin‐Gallausiaux et al. (2024) demonstrated that bacterial metabolites released by *F. nucleatum*, particularly ADP‐heptose, activate the ALPK1/TIFA/NF‐κB signaling axis, resulting in increased IL‐8 expression and induction of anti‐apoptotic programs. These findings suggest that inflammatory signaling triggered by *F. nucleatum* extends beyond immune cell activation and directly contributes to tumor cell survival and adaptation to microenvironmental stress.

Collectively, the available evidence supports a model in which *F. nucleatum* functions as an active immunomodulatory agent capable of remodeling the tumor microenvironment through sustained inflammatory activation, macrophage reprogramming, and immune evasion, thereby fostering conditions permissive to CRC progression.

### 3.3 Epigenetic Regulation and Oncogenic Signaling

Beyond immune modulation, *F. nucleatum* appears capable of directly reprogramming tumor cell behavior through epigenetic and oncogenic mechanisms that favor CRC progression. Experimental evidence suggests that bacterial infection promotes transcriptional and post‐transcriptional alterations capable of sustaining proliferative signaling and enhancing tumor cell survival.

Epigenetic regulation comprises heritable but reversible modifications in gene expression that occur without changes in the underlying DNA sequence. These mechanisms include DNA methylation, histone modifications, chromatin remodeling, and post‐transcriptional RNA regulation, all of which play fundamental roles in controlling cell differentiation, proliferation, and survival. In CRC, epigenetic dysregulation contributes to tumor initiation and progression by altering the expression of oncogenes, tumor suppressor genes, and signaling pathways involved in cell‐cycle regulation, apoptosis, and invasion. Increasing evidence further suggests that microorganisms may influence these regulatory processes by modulating host epigenetic machinery, thereby promoting persistent transcriptional reprogramming and creating a cellular environment favorable to tumor development (Flavahan et al. 2017; Feinberg et al. 2016; Xu et al. 2022). One of the most consistent mechanistic findings involves dysregulation of epigenetic pathways associated with RNA methylation. Using CRC cell lines, murine xenograft models, and complementary human tissue analyses, Xu et al. (2022) demonstrated that increased expression of methyltransferase‐like 3 (METTL3) and enhanced m6A methylation of pri‐miR‐4717‐3p promote microRNA maturation and suppress MAP2K4 expression, thereby favoring CRC cell proliferation and tumorigenicity (Xu et al. 2022). These findings support a model in which *F. nucleatum* contributes to tumor progression by reshaping gene regulatory networks involved in cell growth and oncogenic signaling.

Using CRC cell lines, murine models, and clinical tumor specimens, Li et al. (2024) demonstrated that modulation of the E‐cadherin/β‐catenin/TCF4 axis represents a relevant mechanism linking *F. nucleatum* infection to oncogenic reprogramming. Activation of β‐catenin‐associated signaling pathways has been associated with altered transcriptional responses and enhanced tumor cell adaptability, reinforcing the capacity of *F. nucleatum* to influence molecular programs beyond inflammatory signaling (Li et al. 2024).

Collectively, these findings indicate that *F. nucleatum* may promote colorectal carcinogenesis not only through immune and inflammatory modulation, but also by directly altering epigenetic and transcriptional programs that sustain tumor growth and malignant progression.

Although these findings consistently support a mechanistic role for *F. nucleatum* in epigenetic regulation, most evidence derives from experimental models, with comparatively limited validation in human tissues. Consequently, further translational and clinical studies are needed to determine the extent to which these mechanisms contribute to CRC progression in patients.

### 3.4 EMT, Endothelial Interaction, Invasion, and Metastasis

Experimental evidence consistently supports a role for *F. nucleatum* in promoting metastatic competence through coordinated effects on endothelial interaction, EMT, and inflammatory signaling. Rather than acting through a single pathway, the bacterium appears to amplify pro‐metastatic programs by reshaping tumor–endothelial interactions and enhancing migratory and invasive phenotypes in CRC cells.

One of the principal mechanisms implicated in metastatic dissemination involves activation of the ALPK1/NF‐κB/ICAM1 signaling axis. Upregulation of ICAM1 expression facilitates adhesion of tumor cells to endothelial surfaces, thereby promoting transendothelial migration and vascular extravasation. Using CRC cell lines, endothelial adhesion assays, and murine metastasis models, Zhang et al. (2022) demonstrated that *F. nucleatum* activates the ALPK1/NF‐κB/ICAM1 signaling pathway, resulting in enhanced endothelial adhesion and increased pulmonary metastasis formation, thereby supporting a functional contribution to vascular dissemination. In parallel, bacterial‐induced suppression of miR‐5692a has been associated with increased IL‐8 expression and activation of ERK signaling, thereby promoting EMT and enhancing migratory and invasive behavior in CRC cells (Yu et al. 2025).

These findings indicate that *F. nucleatum* not only facilitates physical dissemination through endothelial adhesion but also actively reprograms cellular phenotypes associated with metastatic progression.

Collectively, the available evidence suggests that *F. nucleatum* promotes CRC dissemination through interconnected mechanisms involving inflammatory signaling, endothelial remodeling, and EMT activation, reinforcing its role as an active driver of tumor aggressiveness and metastatic potential.

### 3.5 Metabolic Reprogramming, Cell Death, and Therapeutic Resistance

Beyond inflammatory and oncogenic signaling, *F. nucleatum* appears capable of promoting tumor adaptation through mechanisms involving metabolic reprogramming, disruption of regulated cell death pathways, and reduced responsiveness to chemotherapy. Emerging evidence suggests that bacterial‐driven metabolic alterations contribute not only to tumor survival but also to therapeutic resistance in CRC.

Ferroptosis is a regulated form of cell death characterized by iron‐dependent accumulation of lipid peroxides to levels that become incompatible with cell survival. Unlike apoptosis or necrosis, ferroptosis is driven by oxidative damage to membrane phospholipids and is tightly controlled by antioxidant defense systems, particularly glutathione peroxidase 4 (GPX4), which detoxifies lipid hydroperoxides and prevents membrane damage. By preventing excessive lipid peroxidation, GPX4 preserves membrane integrity and protects cancer cells from ferroptotic cell death. Increasing evidence indicates that suppression of ferroptosis enables cancer cells to survive oxidative stress, thereby promoting tumor progression and reducing responsiveness to anticancer therapies. Consequently, mechanisms that inhibit ferroptosis have emerged as important contributors to chemoresistance in CRC (Y. Wang, Zhang, et al. 2024).

One of the principal mechanisms implicated in treatment resistance involves suppression of ferroptosis. Using CRC cell lines, murine models, and complementary human tumor analyses, Li et al. (2024) demonstrated that *F. nucleatum* modulates the E‐cadherin/β‐catenin/TCF4 signaling axis, resulting in increased GPX4 expression and reduced oxidative lipid damage, thereby promoting resistance to oxaliplatin treatment. These findings provide a biologically plausible explanation for reduced chemotherapeutic efficacy in tumors with high intratumoral bacterial burden (Li et al. 2024). Additional evidence indicates that bacterial metabolites may further contribute to tumor resilience. Activation of the ALPK1/TIFA/NF‐κB signaling pathway through ADP‐heptose release has been associated with increased anti‐apoptotic signaling and reduced responsiveness to 5‐fluorouracil (5‐FU), suggesting that *F. nucleatum*‐mediated inflammatory activation may indirectly favor chemoresistance (Martin‐Gallausiaux et al. 2024).

Complementing these findings, Zhou et al. (2025) used CRC cell lines, murine models, and multi‐omics analyses of human cohorts to demonstrate that *F. nucleatum* induces metabolic dysregulation associated with lipid accumulation, inflammatory activation, and disruption of intestinal barrier integrity. Importantly, these effects appear partially reversible through microbiome‐targeted interventions, including *Bifidobacterium animalis*, bile salt hydrolase‐producing bacteria, and ursodeoxycholic acid, highlighting potential therapeutic strategies to counteract the pro‐tumorigenic effects of this pathobiont.

Collectively, these findings reinforce the concept that *F. nucleatum* contributes to CRC progression not only through immune and molecular reprogramming but also by facilitating tumor survival under metabolic and therapeutic stress conditions.

**FIGURE 1:**
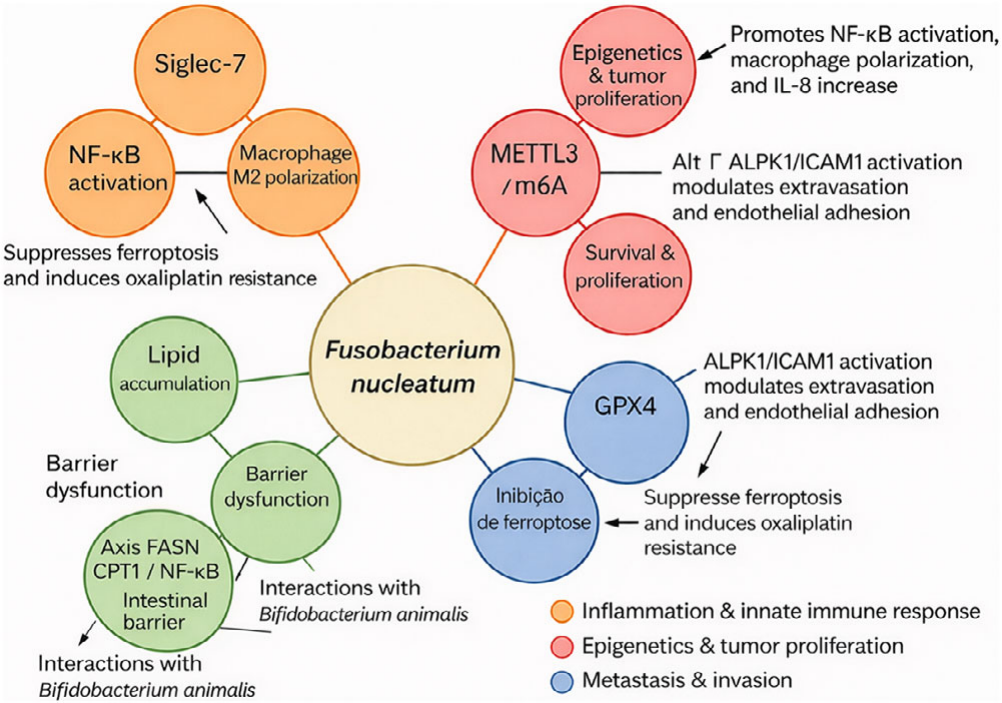
Integrative host–microbe mechanistic framework of *Fusobacterium nucleatum* in colorectal cancer progression.

An integrative conceptual framework summarizing the principal host–microbe mechanistic axes associated with *F. nucleatum* in CRC is presented in Figure 1. A comparative overview of representative experimental models, exposure strategies, and principal mechanistic findings is provided in Table 1.

**TABLE 1:** Representative experimental evidence supporting host–microbe mechanisms of *Fusobacterium nucleatum* in colorectal cancer.

Study (year)	Country	Experimental design	Models used	*F. nucleatum* exposure	Analytical methods	Main mechanistic findings
Lamprinaki et al. 2021	UK/Canada	In vitro	moDCs; monocytes; U937 (Siglec‐7 KO)	*F. nucleatum* ssp. *animalis* ATCC 51191; LPS; MOI 5–50	Flow cytometry, ELISA, Siglec‐Fc binding, Western blot, NMR	Detailed immunological characterization showing direct interaction between *F. nucleatum* LPS/O‐antigen and Siglec‐7, promoting immunomodulation and pro‐tumoral macrophage phenotypes
Xu et al. 2022	China	In vitro + in vivo + tissue analysis	HCT116, LoVo, SW480, HT29; xenograft models	*F. nucleatum* MOI 10–100 for 24 h	qRT‐PCR, Western blot, ChIP‐seq, luciferase assay	Activation of METTL3/m6A/miR‐4717‐3p axis leading to enhanced tumor proliferation and clonal expansion
Zhang et al. 2022	China	In vitro + in vivo	HCT116, HUVECs; murine metastasis model	*F. nucleatum* MOI 100	Western blot, adhesion/transendothelial migration assays	Demonstrates ALPK1/NF‐κB/ICAM1 axis activation, facilitating endothelial adhesion, extravasation, and metastasis
Li et al. 2024	China	In vitro + in vivo + clinical samples	HCT116, HT29; murine models	*F. nucleatum* ATCC 25586; coculture	Western blot, IHC, lipid peroxidation assays (MDA, GSH)	Shows oxaliplatin resistance via suppression of ferroptosis through E‐cadherin/β‐catenin/GPX4 axis
Martin‐Gallausiaux et al. 2024	France	In vitro	HT29, HCT116	*F. nucleatum* (supernatant and ADP‐heptose)	NF‐κB assays, qPCR, Western blot, KO‐TIFA	Evidence of ALPK1/TIFA/NF‐κB activation by bacterial metabolite ADP‐heptose, increasing IL‐8 and anti‐apoptotic signaling
Rezasoltani et al. 2025	Iran	Ex vivo + bioinformatics	Human biopsies (adenoma–CRC)	FadA detected by qPCR (no experimental infection)	qRT‐PCR, ceRNA network analysis	Association of FadA with ANXA2–LINC00460–let‐7a‐2 ceRNA network, suggesting early transcriptional reprogramming
Yu et al. 2025	China	In vitro + in vivo (liver metastasis)	HT29, SW480; murine hepatic metastasis model	*F. nucleatum* MOI 100; 24 h	RNA‐seq, Western blot, qPCR, bioluminescence	Demonstrates EMT and liver metastasis via miR‐5692a/IL‐8/ERK signaling axis
Zheng et al. 2025	China	In vitro	RAW264.7 macrophages	*F. nucleatum* MOI 10–100; 4 h	RNA‐seq, qPCR, Western blot, KEGG/GO	Shows NF‐κB‐dependent M2 macrophage polarization and cytokine–receptor signaling activation
Zhou et al. 2025	China	In vitro + in vivo + omics	CRC cell lines; murine models; human cohorts	*F. nucleatum* infection ± *B. animalis*, *E. coli*‐BSH, UDCA	Metagenomics, metabolomics, WB, qPCR	Links *F. nucleatum* to lipid accumulation, inflammation, and intestinal barrier disruption; effects reversed by biotherapeutic interventions

### 3.6 Translational Implications and Future Perspectives

CRC represents a paradigmatic model of tumor–microbiome interactions in which environmental exposures, host biology, and microbial composition converge to influence disease progression. Although the present review focused primarily on mechanistic experimental evidence, emerging translational frameworks suggest that the biological effects of *F. nucleatum* should be interpreted within a broader context integrating lifestyle factors, dietary exposures, and tumor molecular heterogeneity.

In this regard, the framework of molecular pathological epidemiology (MPE) has increasingly contributed to understanding how long‐term environmental exposures interact with tumor‐associated microbial and molecular features. Rather than considering CRC as a biologically homogeneous disease, MPE approaches integrate host factors, microbial composition, and tumor biomarkers to explain interindividual variability in disease development and progression (Ogino et al. 2011). Such perspectives may be particularly relevant for *F. nucleatum*‐associated CRC, given the heterogeneous distribution of bacterial burden across tumor subtypes and immune contexts.

Recent advances in the prospective cohort incident‐tumor biobank method (PCIBM) further reinforce the value of integrating longitudinal exposure data with tumor microbiome analyses and molecular biomarkers. This framework has been proposed as a powerful strategy for investigating the interactions among environmental exposures, tumor molecular features, and microbiota in precision medicine and population health research (Ogino et al. 2025). In particular, long‐term dietary patterns, including fiber‐rich versus fiber‐poor diets, have been associated with differential prevalence of *F. nucleatum*‐positive colorectal tumors, suggesting that environmental factors may shape microbial colonization and tumor biology over time (Mehta et al. 2017). These observations highlight the importance of investigating how sustained environmental exposures interact with host–microbe mechanisms identified in experimental systems.

From a translational perspective, the mechanistic pathways synthesized in this review support the potential utility of *F. nucleatum* as both a biomarker and a therapeutic target. Detection of bacterial DNA in tumor tissue or fecal samples may contribute to risk stratification and disease monitoring, while microbiome‐directed interventions, including probiotics, prebiotics, bacteriophage‐based approaches, and strategies targeting bacterial virulence pathways, may offer opportunities to improve therapeutic responsiveness. Moreover, the context‐dependent role of *F. nucleatum* in shaping immune responses, including possible interactions with immunotherapy efficacy, reinforces the need for integrative studies combining microbial, molecular, and clinical data (X. Wang, Fang, et al. 2024). Future investigations integrating mechanistic evidence with epidemiological and longitudinal frameworks will be essential to refine personalized approaches at the tumor–microbiome interface.

## 4 Conclusion

*F. nucleatum* emerges as an active biological modulator of colorectal carcinogenesis through coordinated effects on host immune signaling, epigenetic regulation, metabolic adaptation, and metastatic competence. Rather than acting solely as a passive microbial colonizer, accumulating experimental evidence supports its role in reshaping the tumor microenvironment and promoting conditions permissive to tumor progression, immune evasion, and therapeutic resistance.

The mechanistic pathways synthesized in this review highlight the multifaceted nature of host–microbe interactions in CRC. Importantly, growing evidence suggests that the biological effects of *F. nucleatum* may be influenced by tumor heterogeneity and long‐term environmental exposures, reinforcing the importance of integrative translational frameworks.

Collectively, these findings support the potential clinical relevance of *F. nucleatum* as both a biomarker and a therapeutic target while underscoring the need for standardized experimental models and translational studies integrating microbial, molecular, and clinical data to refine precision approaches at the tumor–microbiome interface.

## Funding

This study was funded by the Coordination for the Improvement of Higher Education Personnel (CAPES), Brazil—Finance Code 001.

## Ethics Statement

This study is a systematic review based exclusively on published literature and did not involve the collection of new data from human participants or animals. Therefore, approval by an institutional ethics committee and informed consent were not required.

## Conflicts of Interest

The authors declare no conflicts of interest.

## Data Availability

No new data were generated or analyzed in this study. Data sharing is not applicable to this article as no datasets were created or analyzed during the current study.
